# The vaginal microbiome of pregnant people living with HIV on antiretroviral therapy in the Democratic Republic of Congo: a pilot study and global meta-analysis

**DOI:** 10.1128/msphere.00597-25

**Published:** 2026-01-26

**Authors:** Kimberley S. Ndlovu, Ricardo R. Pavan, Jacqueline Corry, Ann C. Gregory, Samia Mahamed, Natalia Zotova, Martine Tabala, Pelagie Babakazo, Nicholas T. Funderburg, Marcel Yotebieng, Nichole R. Klatt, Jesse J. Kwiek, Matthew B. Sullivan

**Affiliations:** 1 Department of Microbiology, The Ohio State University, Columbus, Ohio, USA; 2 Centre of Microbiome Science, The Ohio State University, Columbus, Ohio, USA; 3 The Infectious Disease Institute, The Ohio State University, Columbus, Ohio, USA; 4 Center for Retrovirus Research, The Ohio State University, Columbus, Ohio, USA; 5 Department of Biological Science, University of Calgary, Calgary, Alberta, Canada; 6 Albert Einstein College of Medicine, Bronx, New York, USA; 7 Kinshasa School of Public Health, Kinshasa, Democratic Republic of Congo; 8 Division of Medical Laboratory Science, School of Health and Rehabilitation Sciences, The Ohio State University, Columbus, Ohio, USA; 9 Department of Surgery, Division of Surgical Outcomes and Precision Medicine Research, University of Minnesota, Minneapolis, Minnesota, USA; 10 Department of Civil, Environmental and Geodetic Engineering, The Ohio State University, Columbus, Ohio, USA; University of Michigan, Ann Arbor, Michigan, USA

**Keywords:** vaginal microbiome, human immunodeficiency virus, antiretroviral therapy, human microbiome, metaanalysis, global health

## Abstract

Recent studies reveal that a suboptimal vaginal microbiome (VMB), including the enrichment of anaerobic bacteria associated with multiple female genital disorders, is linked to adverse pregnancy and birth outcomes in pregnant people. Problematically, however, the majority of the available data, to date, is biased toward highly developed, Global North countries, leaving underrepresented populations like the Democratic Republic of the Congo (DRC) poorly characterized. Here, we investigate the VMB from a cohort of 82 pregnant people living with human immunodeficiency virus (PLWH) on antiretroviral therapy (ART) from the DRC. Specifically, we explore the associations between the VMB via 16S rRNA gene sequencing and maternal peripheral immune factors. Additionally, we compare the VMB of pregnant PLWH-ART from DRC with publicly available VMB data (5 studies, 1861 samples) in a meta-analysis to elucidate the impact of HIV on the VMB. Combined, these analyses revealed the differences in community structure and predicted function of the microbiota between pregnant PLWH-ART and pregnant people without HIV (PWoH). Taxonomically, the VMB of DRC PLWH-ART were enriched for *Lactobacillus iners-*dominated VMBs (53%) or a diverse, polymicrobial VMB, that is, bacterial vaginosis (BV) (43%). Functional predictions made from these taxa suggested that protein-coupled receptors, amino sugar and nucleotide sugar metabolism, fatty acid metabolism, and polycyclic aromatic hydrocarbon degradation pathways were differentially abundant between the communities. Correlation with host plasma immune factors revealed putative links between some VMB metrics (e.g., alpha diversity and species abundance) that have been linked to adverse pregnancy and birth outcomes.

**IMPORTANCE:**

Human immunodeficiency virus (HIV) remains prevalent in sub-Saharan Africa, where it has been linked to adverse birth outcomes. Suboptimal vaginal microbiomes (VMBs) have shown similar links. This pilot study fills critical gaps in understanding how HIV interacts with the pregnant VMB in populations underrepresented in microbiome research, like the Democratic Republic of the Congo (DRC). We identified maternal systemic immune factors associated with suboptimal VMBs that have been linked to poor birth outcomes. In a global meta-analysis, we found significant taxonomic and functional differences in the VMBs between pregnant people living with and without HIV, revealing potential biomarkers that increase the risk of adverse birth outcomes. These findings provide crucial insights into VMB features that may influence pregnancy health in PLWH-ART, guiding future research and tailored interventions to support safer pregnancies in the DRC and similar populations.

This study is registered with NCT03048669.

## INTRODUCTION

Human immunodeficiency virus (HIV) is a lentivirus that targets CD4+ T cells and weakens the immune system. HIV is transmitted through sexual contact, blood, needles, or from mother to infant, leading to chronic systemic infection. In July 2024, Joint United Nations Programme on HIV/AIDS (UNAIDS) estimated that women in Sub-Saharan Africa account for approximately 62% of HIV infections (https://www.unaids.org/en/resources/fact-sheet). Studies show that pregnant PLWH on ART (PLWH-ART) have a higher risk of adverse pregnancy and birth outcomes, including low birth weight (1–3) and preterm birth (PTB) (4–6). These adverse outcomes are also related to the vaginal microbiome (VMB), which, when perturbed toward dysbiosis or bacterial vaginosis (BV), is linked to PTB (7, 8) and factors leading to it like premature rupture of membranes (9, 10), chorioamnionitis (11, 12), and preeclampsia (13).

The VMB is characterized using a community state type (CST) framework, which helps evaluate the combined effects of VMB composition and HIV on pregnancy outcomes. Under this framework, high diversity, non-optimal VMBs, clinically referred to as BV, are classified as CST IV, whereas *Lactobacillus*-dominated, optimal VMBs are classified as CST I, II, III, and V (14). CST III represents a transitional state type that oscillates between optimal and non-optimal (15). Studies have shown that Sub-Saharan African women, particularly those of African ancestry, tend to have CST IV and CST III VMBs, which are associated with increased HIV acquisition (16, 17) and adverse pregnancy and birth outcomes (18). Therefore, understanding how HIV-associated changes to the VMB influence maternal and infant health remains essential.

The impact of HIV on the VMB remains unclear. Studies report changes in composition and CST among PLWH-ART (19, 20), while others show no significant differences PWoH (21, 22). Despite these conflicting results, other findings are more consistent, for example, CST IV and HIV-associated CVMBs are linked to increased genital inflammation and marked by elevated vaginal proinflammatory cytokines and chemokines (23, 24). In pregnant PLWH-ART, these inflammatory phenotypes are associated with adverse pregnancy and birth outcomes, including PTB (25–27). Systemic inflammation in PLWH-ART is also documented (28, 29) and is linked to adverse health outcomes like cardiovascular disease (30, 31).

To date, no study has investigated the relationship between the VMB and the soluble systemic immune factors in pregnant PLWH-ART. Here, we present an exploratory, pilot study of the VMB from pregnant PLWH-ART in Kinshasa, DRC, to identify VMB-associated circulating immune factors. Furthermore, we contextualize the results through a meta-analysis that includes VMB data sets from other pregnant populations, providing a broader perspective on VMB-HIV interactions. Finally, we discuss how these findings relate to pregnancy and birth outcomes in PLWH-ART.

## RESULTS AND DISCUSSION

### Study population, sampling strategy, data generation, and meta-analysis

**Fig 1:**
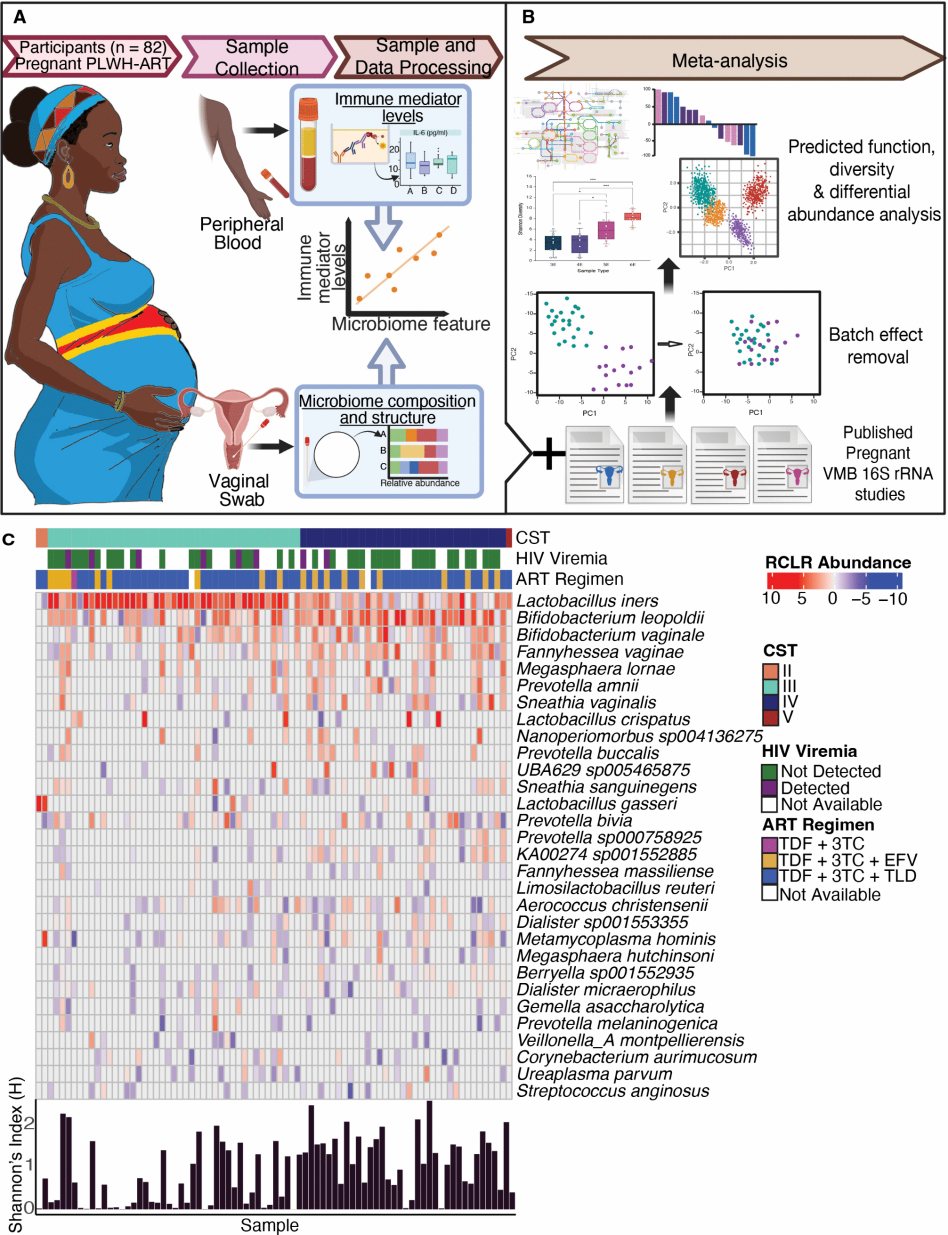
Study schematic and VMB composition of the CQI-PMTCT cohort. (**A**) Study schematic of the CQI-PMTCT study. Peripheral blood and vaginal swabs were collected from pregnant PLWH-ART in the DRC and processed in Columbus, OH. (**B**) VMB data from the CQI-PMTCT cohort were combined with other publicly available pregnant VMB data in a meta-analysis. (**C**) Heatmap of RCLR abundances of the top 30 most abundant microbial taxa from 81 women. CSTs were determined using VALENCIA. HIV viremia, ART regimen, and per sample Shannon Index values are shown. RCLR, centered log-ratio; PLWH-ART, people living with HIV on antiretroviral therapy; CQI-PMTCT, Continuous Quality Improvement prevention of mother-to-child transmission; CST, community state type.

To date, no study has investigated the VMB of people in the DRC. In this pilot study, we generated amplicon sequencing data to investigate the VMB of pregnant PLWH-ART in the Continuous Quality Improvement prevention of mother-to-child transmission (CQI-PMTCT) cohort and its association with the systemic immune system (32, 33). We collected vaginal swabs and peripheral blood from 82 pregnant PLWH-ART in Kinshasa, DRC, to generate 16S rRNA gene data and plasma immune factor levels (see Materials and Methods, Fig. 1A). Participant characteristics by CST, including participant age, gestational age, ART regimen, gravidity, socioeconomic status, and adverse birth outcomes, are summarized in Table 1. This cohort included only pregnant PLWH-ART; therefore, we also conducted a meta-analysis integrating VMB data with other published pregnant VMB data sets to compare PLWH-ART and PWoH (Fig. 1B).

**TABLE 1:** Participant demographics at enrollment stratified by community state type^a^

	No. of participant (%)^b^ with characteristic		
Characteristic	All (*n* = 81)	CST III (*n* = 43)	CST IV^c^ (*n* = 35)
Median age in years (IQR^d^)	32 (28–35)	33 (30–34)	30 (26–35)
HIV-1 viremia			
Detected (> 40 copies/mL)	8 (10)	6 (14)	2 (6)
Not detected (≤ 40 copies/mL)	42 (52)	20 (47)	19 (54)
Missing	31 (38)	17 (40)	12 (34)
Gestational age (weeks)			
Second trimester (13–26)	28 (35)	14 (33)	13 (37)
Third trimester (27+)	32 (40)	19 (44)	11 (31)
Missing	21 (26)	10 (23)	11 (31)
ART regimen**^e^**			
TDF + 3TC	1 (1)	1 (2)	0 (0)
TDF + 3 TC + EFV	19 (23)	9 (21)	10 (29)
TDF + 3 TC + TLD	59 (73)	32 (74)	24 (69)
Missing	2 (2)	1 (2)	1 (3)
Gravidity			
Primigravida	13 (16)	5 (12)	8 (23)
Multigravida	68 (84)	38 (88)	27 (77)
Adverse birth outcome (preterm birth, stillbirth, low birth weight)			
Yes	22 (27)	12 (28)	8 (23)
No	56 (67)	26 (60)	26 (74)
Missing	3 (6)	5 (12)	1 (3)
SES quartile**^f^**			
1 (lowest)	1 (1)	0 (0)	1 (3)
2	15 (19)	9 (21)	6 (17)
3	37 (46)	20 (47)	14 (40)
4 (highest)	28 (35)	14 (33)	14 (40)

^a^ CST, community state type.

^b^ Percentages rounded off to the nearest whole number.

^c^ CST IV combined subtypes A (*n *= 4), B (*n *= 6), and C (*n* = 25). Statistics of three participants in CST II (*n *= 2) and CST V (*n *= 1) are not reported.

^d^ IQR, interquartile range.

^e^ ART, antiretroviral therapy; 3TC, lamivudine; TDF, tenofovir disoproxil fumarate; EFV, efavirenz; TLD, tenofovir/lamivudine/dolutegravir.

^f^ SES, socioeconomic status.

### In the CQI-PMTCT cohort, CST III and CST IV types are predominant in the VMB of pregnant PLWH-ART in the DRC

Analysis of the top 30 most abundant species showed that 53% of participants (*n* = 43) were dominated by *Lactobacillus iners*, while 43% (*n* = 35) were dominated by a polymicrobial anaerobic consortium (*Bifidobacterium leopoldii*, *Bifidobacterium vaginae*, *Fannyhesea vaginae*, *Prevotella* spp., and UBA629 sp0054587) (Fig. 1C). These profiles classify the CVMBs of pregnant PLWH-ART as CST III *(L.iners)* or CST IV (polymicrobial consortium).

**Fig 2:**
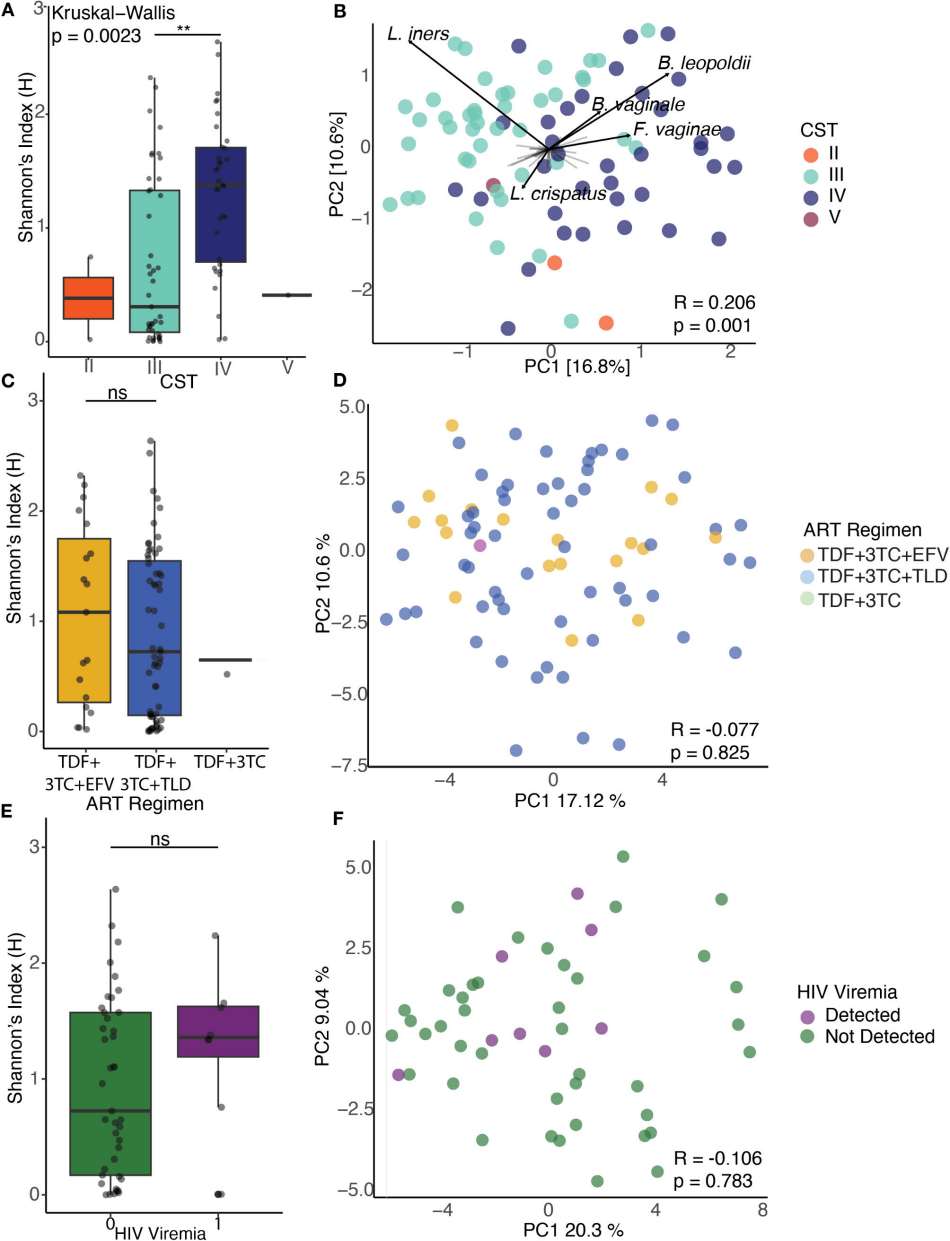
Ecological statistics of the VMB in the CQI-PMTCT cohort (**A**). Shannon entropy diversity stratified by CSTs (Kruskal-Wallis *P* =0.00234). (**B**) PCA biplot of Robust Aitchison distances colored by CSTs (ANOSIM, R = 0.206, *P* = 0.001). The loadings of the top five most abundant species across all samples are labeled to highlight drivers of variation among CST clusters with black arrows to indicate the magnitude and directionality. (**C**) Shannon indices aggregated by ART regimen (Kruskal-Wallis *P* = 0.695). (**D**) PCA of Robust Aitchison distances colored by ART regimen for 79 participants (ANOSIM R =−0.077, *P* = 0.825). (**E**) Aggregated Shannon entropy values of HIV viremia for 50 participants (Wilcoxon test *P* = 0.2853). (**F**) PCA of robust Aitchison distances colored by detected viremia (ANOSIM R= −0.106, *P* = 0.783). Within each boxplot, the horizontal lines denote median values and the boxes extend from the 25th to the 75th percentiles. The vertical lines in each box plot show the values with 1.5 IQR. **P < 0.01, ns > 0.05. PCA, principal component analysis. Analysis of similarities (ANOSIM).

Diversity analyses revealed two main patterns. First, within-sample diversity differed significantly across CSTs (*P* = 0.0023, Kruskal-Wallis) (Fig. 2A), consistent with CST IV being more diverse than *Lactobacillus*-dominated CSTs (14). Second, beta diversity showed clustering by CST (analysis of similarities [ANOSIM], R = 0.206, *P* = 0.001, Fig. 2B), with *L. iners* driving variation within CST III and *Bacterial vaginale, B. leopoldii,* and *Fannyhessea vaginae* driving the variation within CST IV.

These results align with studies showing that Sub-Saharan African women are more likely to harbor high-risk, non-optimal CST IV VMB (16, 34, 35). Similar cohorts in geography and size have shown that pregnant PLWH-ART have VMBs dominated by *Gardnerella* spp*.* and *L. iners* (20, 27)*.* Although *L. iners* is common in VMBs of women in Sub-Saharan Africa and worldwide, it is considered less optimal than *Lactobacillus crispatus* or *Lactobacillus jensenii* (14, 34).

Despite its global prevalence, the role of *L. iners* in vaginal health and pregnancy outcomes remains ambiguous. In pregnant PLWH-ART, *L. iners*-dominated VMBs have been linked to increased risk of PTB (20, 27), whereas in pregnant PWoH, *L. iners* has been associated with a lower risk of PTB (36, 37). These contrasting findings highlight the complexity of *L. iners. L. iners* often coexists with non-optimal, CST IV bacteria, is less stable in the vaginal microbiome, and so can facilitate transitions to CST IV VMBs (38). *L. iners* also shows pathogenic potential, producing the less protective L-lactic acid isomer (39–41), and expressing the homologs of cholesterol-dependent cytolysins found in other pathogens but not in other *Lactobacilli* (42–44). However, its presence even in healthy, asymptomatic individuals and its strain diversity demonstrate that *L. iners* functions as a vaginal symbiont capable of persisting alongside more protective *Lactobacilli* (45, 46). Further strain-resolved studies are needed to clarify the complex roles of *L. iners* in vaginal health and pregnancy.

The other two most abundant bacteria in the CQI-PMTCT cohort, *B. leopoldii* and *B. vaginale,* both formerly *Gardnerella vaginalis* (47), are commonly found at high abundance in Sub-Saharan populations (20, 27), even in healthy, asymptomatic VMBs (48–50). These CST IV bacteria have been associated with adverse pregnancy outcomes like PTB (7, 51) and fetal death (52–54). Overall, our results align with studies showing the VMBs of Sub-Saharan African PLWH-ART are non-*Lactobacillus*-dominated (CSTs IV) or *L. iners*-dominated (CST III) VMBs, which may contribute to higher rates of HIV, sexually transmitted infections (STIs) (55, 56), and PTB (19, 20).

### VMB structure is not associated with HIV viremia or ART regimen in the CQI-PMTCT cohort

Next, we sought to investigate clinical characteristics that might influence the VMB in the CQI-PMTCT. We focused particularly on the type of ART regimen and HIV viremia. Currently, there are inconclusive results on whether ART impacts the VMB (57–59). The same is true for HIV viremia, particularly in chronic HIV infection (20, 21, 60). Therefore, we asked whether the type of ART regimen or HIV viremia in pregnant PLWH-ART had an influence on the VMB. We hypothesized that the type of ART would impact the VMB and that participants with detectable HIV viremia (*n* = 8, Table 1) would have more diverse VMB, with communities that were distinct from those with undetectable HIV viremia (*n* =42, Table 1).

We found no significant differences in alpha diversity at the species level between ART regimen (Kruskal-Wallis test, *P* = 0.695, Fig. 2C) and no differences in VMB structure between the types of ART regimen (ANOSIM R= −0.077, *P* = 0.825, Fig. 2D). We also did not see differences in alpha diversity between detected and undetected HIV viremia (Wilcoxon test, *P* = 0.285, Fig. 2E) and in beta diversity (ANOSIM R = −0.106, *P* = 0.783, Fig. 2F).

A study with a similar cohort in size and geography has shown that ART use did not impact the VMB of Ugandan PLWH-ART (57). However, VMBs have been associated with varying concentrations of ART in the female genital tract and plasma, which means that there is a possibility that long-term ART use could have some influence in the VMB (61). Currently, it is known that topical ARTs, like PrEP and tenofovir, are metabolized by CST IV vaginal bacteria, thereby reducing their efficacy (62–64), but these ARTs do not seem to alter the VMB composition and structure (57–59). While further research might be necessary to investigate the link between ART use and VMB composition, our results contribute to the growing knowledge of the impact, or lack thereof, between ART use and the VMB (65).

Studies have shown that there are no differences in vaginal communities due to HIV viremia or status (21, 60). Conversely, other 16S rRNA gene studies have shown that HIV-associated VMBs have a lower abundance of *Lactobacillus* species, but there was no conclusion on how VMB structure (i.e., beta diversity) is different (56, 66, 67). Curiously, there is inconclusive evidence on how HIV influences VMB composition. On one hand, vaginal bacteria have been shown to interact with the HIV virus or host cells to increase the risk of contracting HIV. For example, CST IV bacteria like *G. vaginalis* induce a local inflammatory response and reduce vaginal epithelial cell barrier and integrity, hence allowing bacterial and viral pathogens, like HIV, to infect the host (16, 68, 69). A recent study also showed that bacterial lectins specific to O-glycans bind to the HIV-1 virion and envelope glycoproteins to increase HIV infectivity and resistance to antibodies (70). However, the mechanisms by which HIV directly impacts bacterial physiology and metabolism remain undefined and unexplored. It is possible that the HIV viral load in the vagina is sufficiently suppressed to permit a detectable interaction between the virus and the vaginal bacteria (71, 72); however, studies on this subject are highly limited. The ambiguity of the impact of chronic and systemic viral sexually transmitted infections like HIV on the VMB remains to be fully explored; however, based on our results for the CQI-PMTCT cohort, we can tentatively conclude and recapitulate the findings of studies that state HIV viremia does not influence the VMB (21, 22, 60). The high percentage of people with undetectable HIV viremia is a testament to the effectiveness of ART use in this cohort.

### Meta-analysis reveals that VMBs are distinct between pregnant PLWH-ART and pregnant PWoH globally

To better understand the role of HIV on the VMB, we integrated this study with five other publicly available pregnant VMB data sets for comparison. To reduce batch effects, data from studies were selected to be as similar to the CQI-PMTCT VMB data as possible (see Materials and Methods and Fig. S1).

**Fig 3:**
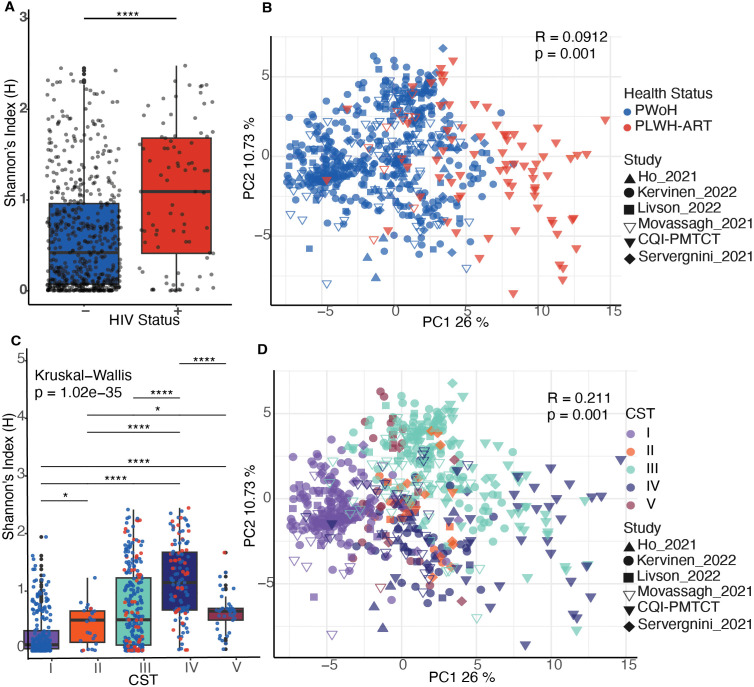
Ecological statistics of the integrated data sets. (**A**) Shannon entropy values between VMBs of pregnant PWoH and PLWH-ART separated by study (Wilcoxon test *P* = 2.661e–07). (**B**) PCA of Aitchison distances showing different bacterial communities by health status (ANOSIM R = 0.518, *P* = 0.001). (**C**) Shannon entropy values stratified by CST (Kruskal-Wallis *P* = 1.023e–35). (**D**) PCA of Aitchison distances showing different bacterial communities by CST (ANOSIM R = 0.399, *P* = 0.001). *P < 0.05, ****P < 10^−4^. PWoH, people without HIV.

After adjusting for study-specific batch effects using partial least square discriminant analysis (PLSDA), we found that within-sample species diversity was significantly higher (Wilcoxon test *P* = 2.661e–07) in samples from pregnant PLWH-ART compared with pregnant PWoH (Fig. 3A). Additionally, VMB community structure was significantly different between pregnant PLWH-ART and pregnant PWoH (ANOSIM R= 0.518, *P* = 0.001, Fig. 3B). When stratified by CST, within-sample species diversity was highest in CST IV samples, which is suboptimal in the VMB (Fig. 3C), and VMB community structure was different by CSTs (ANOSIM R= 0.399, *P* = 0.001, Fig. 3D). These findings indicate that bacterial communities differ between pregnant PLWH-ART and pregnant PWoH.

**Fig 4:**
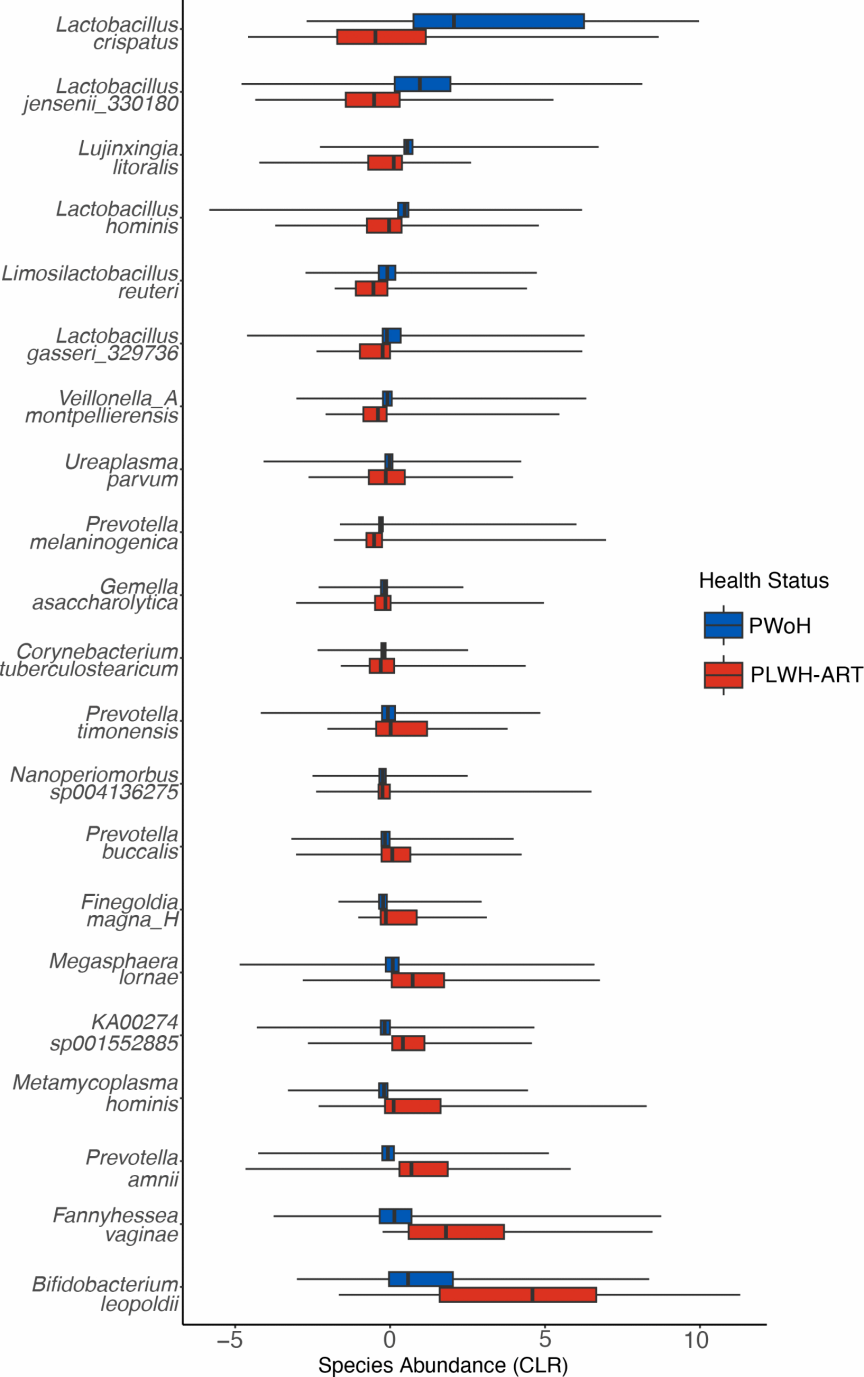
Boxplots showing differentially abundant species in pregnant PLWH-ART and PWoH as determined using three differential abundance analysis methods. A species was considered differentially abundant if it met two of the following criteria: *P*-adjusted value < 0.05 in LinDA; q-value < 0.05 in ANCOM-BC; or effect size > 0.5 in ALDEx2. Within each box, the vertical lines denote median values, and the boxes extend from the 25th to the 75th percentiles. The horizontal lines in each box plot show the values with 1.5 IQR.

To further investigate this claim, we used differential abundance analyses to identify bacterial species that were differentially enriched between the two groups. A species was deemed differentially abundant when it met two of these criteria: *P*-adjusted value < 0.05 in LinDA; q-value < 0.05 in ANCOM-BC; or effect size > 0.5 in ALDEx2. We found that 21 vaginal bacterial species were differentially abundant between the pregnant PLWH-ART and pregnant PWoH groups (Fig. 4). *Lactobacilli* spp*.,* that is, beneficial bacteria like *L. crispatus, Lactobacillus gasseri, Lactobacillus hominis,* and *L. jensenii,* and *Limosilactobacilus reuteri* were more abundant in the VMB of pregnant PWoH. In contrast, the pregnant PLWH-ART group had a high abundance of CST IV, BV-associated bacteria compared with the pregnant PWoH group. For example, species in the *Bacteroidaceae* family, particularly *Prevotella amnni* and *Prevotella timonensis* spp*.* were more abundant in the pregnant PLWH-ART group. Other CST IV-associated bacteria that were more abundant in the pregnant PLWH-ART group were *B. leopoldii, M. lornae, KA00274 sp001552885* (AKA *Amygdalobacter nucleatus*), *F. magna, F. vaginae, M. hominis,* and *Corynebacterium tuberculostearicum* (Fig. 4).

Other studies have found *Lactobacillus spp.* to be more dominant in PLWH not on ART compared with those on ART, regardless of pregnancy state (56, 66, 67). This is corroborated by the CST IV, non-optimal bacteria being more differentially abundant in the pregnant PLWH-ART group compared with the pregnant PWoH. It is possible that this differential abundance in (non)-*Lactobacillus-*dominated VMBs between pregnant PLWH-ART and PWoH existed prior to and during HIV acquisition, as such differences are associated with the risk of HIV acquisition (73). For that reason, these communities may have remained stable in their respective states since. Indeed, one study determined that in 79.7% of VMBs from two longitudinal cohorts (15, 74), CSTs were predominantly mono-stable and rarely transitioned to completely different CSTs, which could explain the persistence of these bacteria in the VMB even after HIV acquisition (38). It is also possible that the influence of sociocultural, environmental, and economic factors (for which self-reported racioethnicity is often a suboptimal proxy (75, 76)), maternal age, and geography (Fig. S2) distorts the impact of HIV status on the VMB in this meta-analysis, as previously observed (48, 49, 77, 78). Additionally, it is important to note that in the cohorts studied to date, CST III and CST IV VMBs, which are linked to an increased risk of HIV acquisition (16, 19, 73, 79, 80), are commonly observed in Sub-Saharan African women; as there was only one other Sub-Saharan cohort in our analysis, these differences are interpreted with caution. Nevertheless, we tentatively conclude that HIV infection is associated with changes in the VMB of pregnant people, as seen in a recent study with similar populations (81). However, future research will have to explore the causative associations of HIV on the pregnant VMB and how this alters pregnancy outcomes.

### Predicted metabolic pathways were differentially abundant among CSTs in the CQI-PMTCT cohort

The CST framework classifies the VMB based on taxonomic composition (14). However, even when microbial communities differ in their taxonomic composition, they might have functional redundancy (82) or, conversely, diverse functional capabilities in the same species (83). Hence, functional prediction of the VMB may yield more specific insights into potential phenotypic differences between CST and, thus, host responses. Here, we predicted the function of the VMB, stratified by CST, using PICRUSt2 based on the KEGG (Kyoto Encyclopedia of Genes and Genomes) database (84, 85). We predicted 6,466 KEGG Orthologs (KOs), which were assigned to 162 KEGG pathways. Of note, these are predicted functions and not direct readouts by the bacterial species.

**Fig 5:**
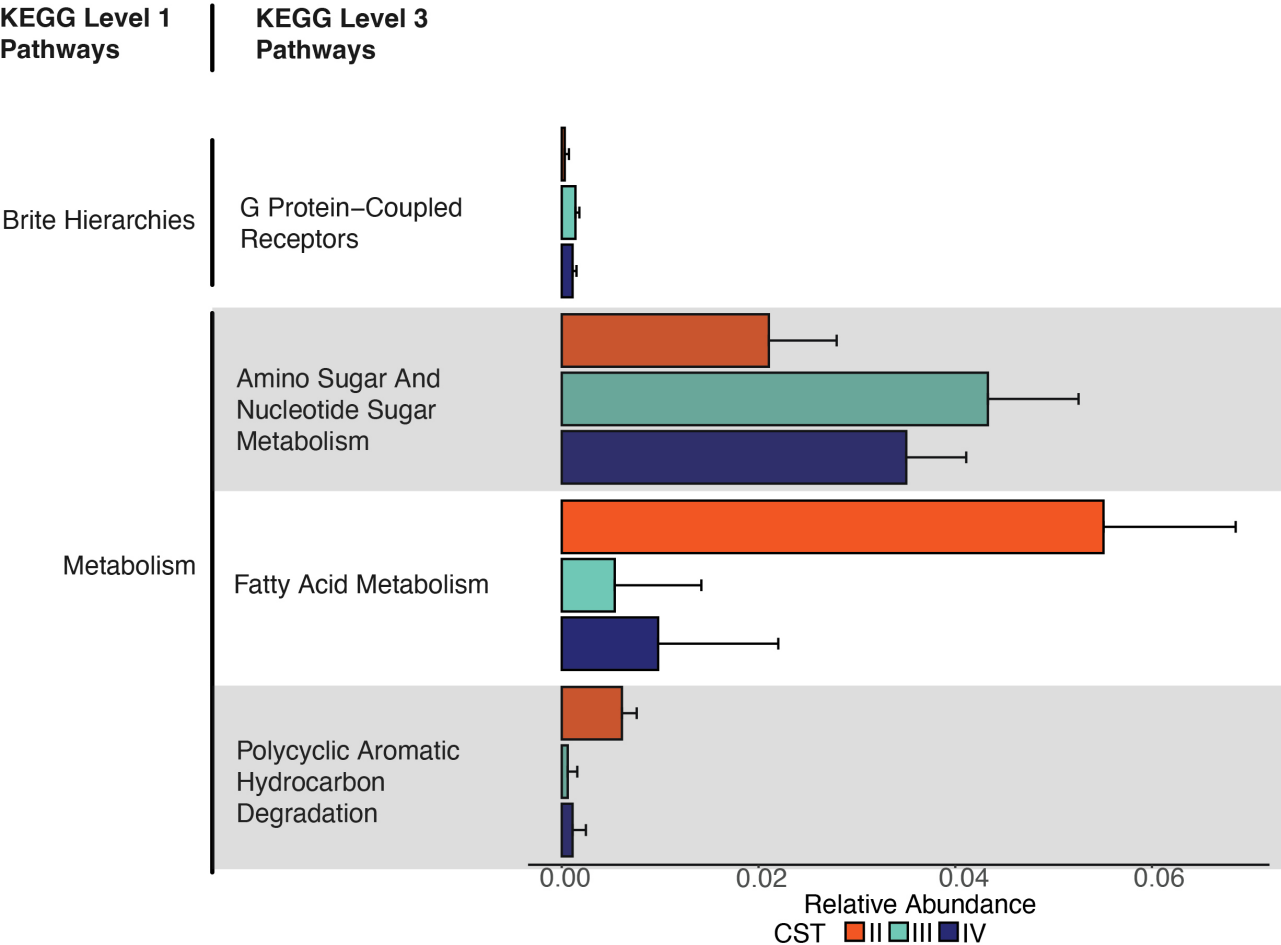
Predicted KEGG pathways differentially abundant between CSTs in the CQI-PMTCT cohort as determined using three differential abundance analysis methods. A pathway was considered differentially abundant if it met two of the following criteria: *P*-adjusted value < 0.05 in LinDA; q-value < 0.05 in ANCOM-BC; or effect size > 0.5 in ALDEx2. Error bars represent mean values plus standard deviation.

Using multiple differential abundance analyses (see Materials and Methods), we identified four KEGG pathways that were differentially abundant between CSTs (Fig. 5), namely G protein-coupled receptors (GPCRs, KO04030), amino sugar and nucleotide sugar metabolism (KO00520), fatty acid metabolism (KO01212), and polycyclic aromatic hydrocarbon degradation (KO00624). Fatty acid metabolism and polycyclic aromatic hydrocarbon degradation had higher relative abundance in CST IV compared to CST III, with the highest abundance of both in CST II. G protein-coupled receptors and amino sugar and nucleotide sugar metabolism were more abundant in CST III, followed by CST IV and CST II (Fig. 5).

Because there were only two samples assigned to CST II, we will focus our discussion on CST III and IV only. A combination of predicted VMB genotypes suggests a plausible mechanism for the disruption of host homeostasis. Short-chain fatty acid (SCFA) metabolism was more abundant in CST IV compared to CST III VMBs, indicating a greater presence of bacteria capable of producing SCFA. CST IV bacteria play a large role in SCFA production, and the increased presence of SCFAs like propionate, acetate, and butyrate has been associated with vaginal dysbiosis (86). Furthermore, the production of SCFA leads to decreased *Lactobacillus* abundance and increased pH in the vagina (86–88). Based on this, we can posit that there were high concentrations of vaginal SFCAs in this cohort. GPCRs were slightly, yet still significantly, more abundant in CST III compared to CST IV CVMSs. GPCRs mediate the immunoregulatory effects of SFCAs (87), and specifically, GPCRs 41, 43, and 109A can inhibit histone deacetylases (HDACs), which subsequently induce a cellular and humoral immune response (87, 89). Simultaneously increased SFCAs and decreased GPCRs may induce a local pro-inflammatory response in this cohort. Given that inflammation in the vaginal tract is associated with adverse birth outcomes, like PTB, in pregnant PLWH-ART (20), which may be linked to increases in SFCA production and decreases in the immunoregulators in the VMB.

Amino sugar and nucleotide sugar metabolism is another pathway that might be implicated in birth outcomes in pregnant PLWH-ART. For bacteria, these are core intermediates involved in peptidoglycan and lipopolysaccharide, among other macromolecule, biosynthesis, energy metabolism, and other processes. In this cohort, this pathway was more abundant in CST III compared with CST IV VMBs. One study found that amino sugar and nucleotide metabolism were negatively associated with mammalian target of rapamycin (mTOR), a kinase that regulates cell growth and proliferation (90). In the same study, mTOR was negatively correlated with *L. iners* and positively correlated with polymicrobial, CST IV bacteria (90). While some of this association may be due to major structural features of the VMB—that is, the presence of a lipopolysaccharide layer of some members but not others—these metabolic products from the VMB of pregnant PLWH-ART may interact with the mTOR pathway, activating it (by CST III bacteria) or inhibiting it (by CST IV bacteria). This interaction could maintain or disrupt vaginal epithelial integrity, potentially determining whether birth outcomes are adverse or normal through a proinflammatory or anti-inflammatory response and either increasing or decreasing the risk of STIs.

Finally, polycyclic aromatic hydrocarbon (PAH) degradation was also differentially more abundant in CST IV than in CST III VMBs. PAHs, which are ubiquitous in the environment, can impact the VMB (8), which can lead to adverse birth outcomes like intrauterine growth restrictions and PTB (91, 92). The polymicrobial state type, CST IV, tends to have varying abundances of putative PAH-degrading bacterial genera, including *BVAB2* and *Megasphaera, Sphingomonas*, *Acinetobacter*, *Micrococcus*, *Pseudomonas*, and *Ralstonia,* and two of the genera, *Megasphaera* and *Pseudomonas,* are present in the CQI-PMTCT cohort (93–95). Therefore, somewhat unexpectedly, a more diverse, polymicrobial CST IV VMB may be better able to degrade PAHs and might offer some protection against their harmful effects. Perhaps, because the Sub-Saharan African region is exposed to a substantial amount of PAHs (96, 97), a CST IV VMB might be advantageous and help explain its prevalence in Sub-Saharan African populations (16, 49, 98). However, a multidisciplinary research study, across life and social sciences, would need to be conducted to investigate this claim (75, 99), which is an important next step. Overall, these findings provide the direction for further studies to clarify these differences with more granularity and the role of bacterial metabolic pathways on reproductive and birth outcomes in pregnant PLWH-ART in vulnerable and underrepresented populations. Additionally, future multi-omics studies should be conducted to progress the CST framework from taxonomy-only-based classification to microbial function and taxonomy-based classification.

### Meta-analysis reveals that predicted metabolic pathways were differentially abundant between pregnant PLWH-ART and pregnant PWoH

**Fig 6:**
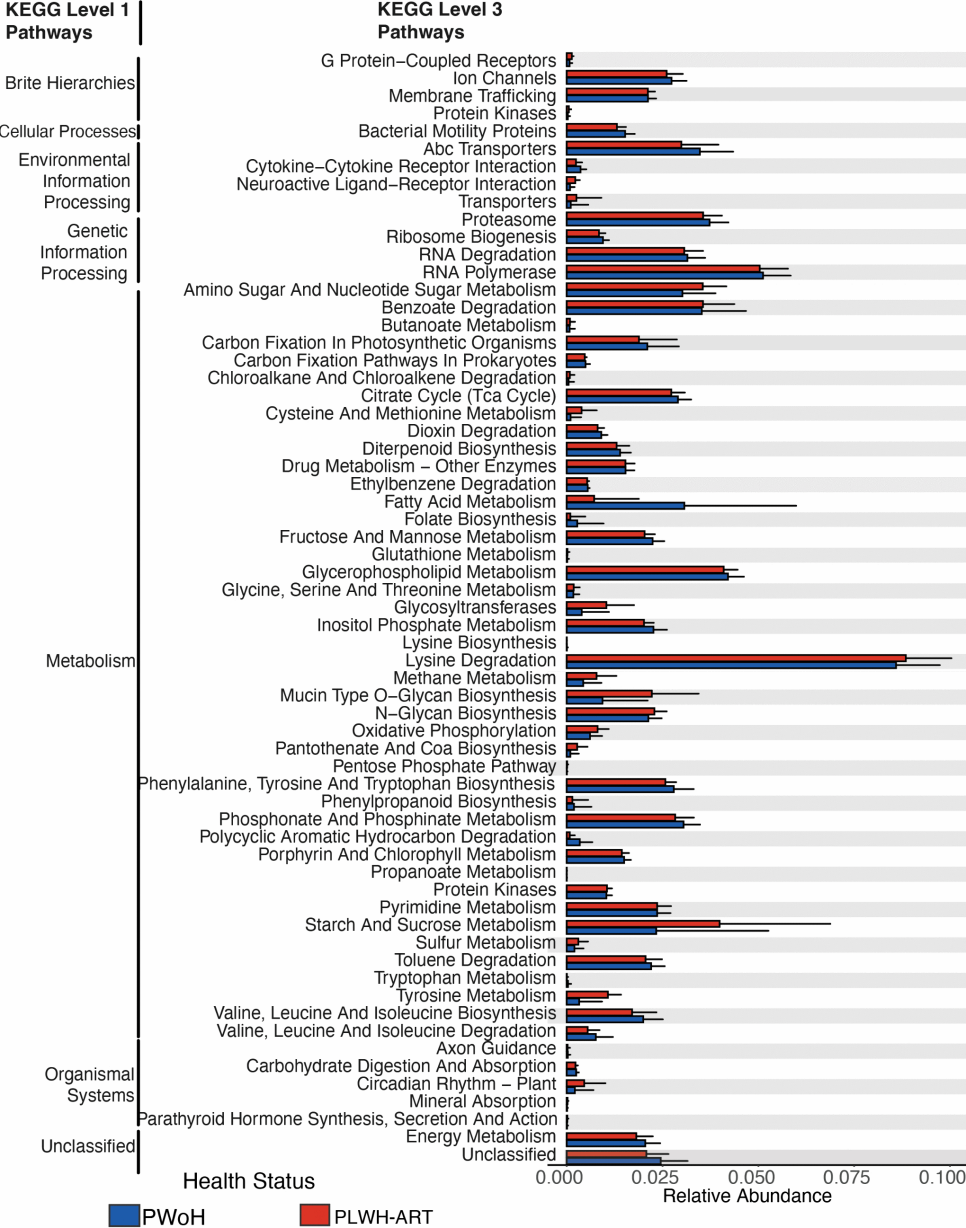
KEGG pathways differentially abundant between pregnant PLWH-ART and pregnant PWoH as determined using three differential abundance analysis methods. A pathway was considered differentially abundant if it met two of the following criteria: *P*-adjusted value < 0.05 in LinDA; q-value < 0.05 in ANCOM-BC; or effect size > 0.5 in ALDEx2. Error bars represent mean values plus standard deviation.

We used PICRUSt2 to predict 4,534 KOs, which were assigned to 130 KEGG pathways. Of those, 62 KEGG pathways were differentially abundant between pregnant PWoH and PLWH-ART VMBs (Fig. 6).

Most of these pathways were primarily involved in metabolism. This could indicate that HIV-associated changes to the pregnant VMB may alter microbial function, which could influence pregnancy and birth outcomes and susceptibility to other vaginal infections in PLWH-ART. For this study, we will focus on only a few pathways that might have relevance to HIV, pregnancy, and birth outcomes. Lysine degradation (KO00310) was differentially more abundant in the pregnant PLWH-ART group compared with the pregnant PWoH group. Products of lysine degradation, cadaverine, and pipecolate were increased in vaginal fluids of women with BV (100). BV, indicative of CST IV, has been associated with adverse birth outcomes in PLWH-ART (19, 20, 27) and increased risk of HIV acquisition (16). Cysteine and methionine metabolism (KO00270) was differentially more abundant in the pregnant PLWH-ART group compared with the PWoH group, and the opposite is true for folate biosynthesis (KO00790). Levels of cysteine in maternal plasma or vaginal fluid have been associated with adverse pregnancy and birth outcomes (101–103). These findings provide direction for further research to investigate how and if HIV-associated changes to the VMB function impact pregnancy and birth outcomes. One pathway, mucin O-glycan synthesis (KO00512) was differentially more abundant in PLWH-ART, and this has been shown to increase the infectivity of sexually transmitted viral pathogens like HIV (70). Future research should investigate how this pathway is involved in pregnancy and birth outcomes in PLWH-ART.

### VMB characteristics are correlated with some plasma immune factors in the CQI-PMTCT cohort

Next, we asked if there was an association between plasma immune factors and the VMB in the CQI-PMTCT cohort. The correlation and causative associations between VMB and vaginal cytokines and chemokines have been well studied (16, 23, 68, 104). Few studies have identified the link between the systemic immune system and the VMB in pregnant PLWH-ART. To this end, we used linear models to identify the VMB metrics (Shannon diversity, CST, and bacterial species abundance) that were associated with the systemic immune system. We hypothesized that CST IV bacteria or highly diverse VMBs would be associated with plasma immune factors that are linked to adverse birth outcomes.

**Fig 7:**
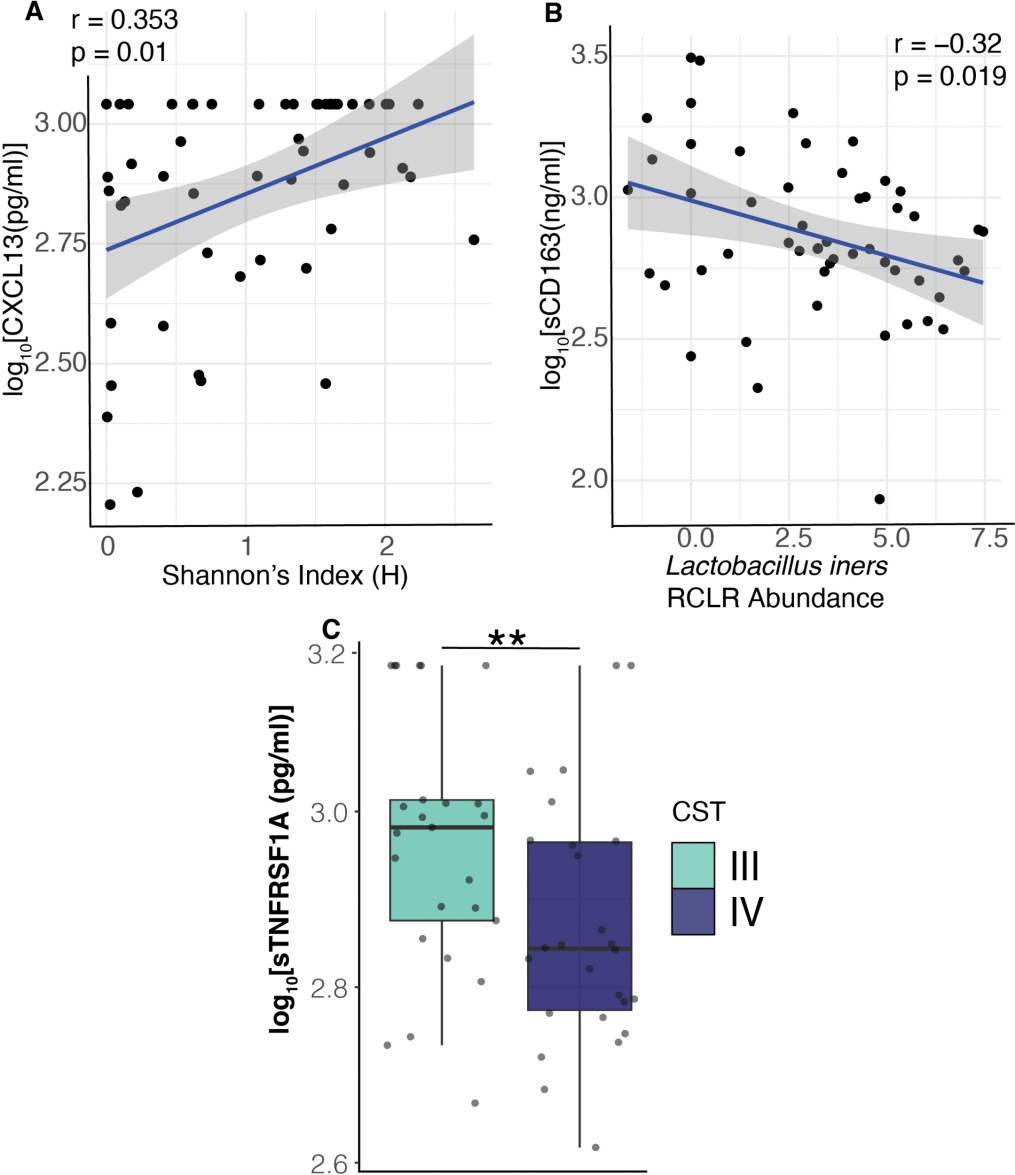
Correlations of VMB metrics to plasma immune mediators. Spearman correlation between (**A**) log_10_-adjusted concentration of CXCL13 to Shannon diversity and (**B**) log_10_-adjusted concentration of sCD163 to *L. iners* abundance. (**C**) Comparisons of log_10_-adjusted concentration of sTNFRSF1A between CST III and IV (Wilcoxon test *P* = 0.0188). Unadjusted two-sided *P*-values reported. Linear regression lines of **A and B** are shown in blue, and the shaded regions represent 95% confidence intervals. Whiskers in **C** show the values with 1.5 IQR. Immune factors abbreviations are listed in Table S3 **P < 0.01.

Shannon diversity was positively correlated with plasma CXCL13 (r = 0.353, *P* = 0.01) (Fig. 7A). When looking at individual taxa, *L. iners* was negatively correlated with sCD163 (r = −0.32, *P* =0.019) (Fig. 7B). In contrast, sTNFRSF1A concentrations were significantly higher in individuals with the CST III VMBs vs those with CST IV VMBs (Fig. 7C). However, in most cases, CST IV associated bacteria were positively correlated with immune markers of inflammation (Fig. S3, Table S8).

Genital inflammation, linked to vaginal dysbiosis, has been associated with adverse pregnancy and birth outcomes (20, 105, 106). Here, we investigate whether systemic inflammation is also linked to the VMB. CXCL13 was positively correlated with Shannon diversity. Abnormal concentrations of CXCL13 have been associated with placental inflammation (107) and PTB (108). Similarly, adverse pregnancy and birth outcomes have been linked with highly diverse VMBs, particularly those with CST IV communities (7, 13, 20, 27). Altogether, this suggests that there is a complex interplay between the VMB and systemic inflammation in pregnant PLWH-ART that impacts pregnancy and birth outcomes.

*L. iners* was negatively correlated with sCD163 (Fig. 7B), an immune factor that has also been linked to PTB (109) and is a predictor of mortality in pregnant PLWH-ART (110, 111). However, one study showed that sCD163 concentrations varied during pregnancy in PLWH-ART, declining in the first trimester and then remaining stable (112). All of this suggests the role vaginal bacteria have in modulating the systemic immune system in pregnant PLWH-ART, and therefore, pregnancy and birth outcomes are complex and may depend on the host’s immunological state. Indeed, the impact gut bacteria have on the systemic immune system might greatly confound the effect of vaginal bacteria on the systemic immune system (113–115). Given that the local vaginal immune profile is distinct from the systemic immune profile, vaginal bacteria may have very little effect on the systemic immune profile (116). However, these associations between the plasma immune factors and maternal VMB may be important for understanding the biological mechanisms underlying HIV-associated pregnancy and associated infant complications.

### Limitations, future opportunities, and conclusions

Our study was limited by the lack of a pregnant PWoH comparison group. Because this work was nested within the larger CQI-PMTCT longitudinal study evaluating long-term ART effects, samples were collected only from pregnant PLWH-ART (32). Despite this limitation, our study provides important details about the VMB of pregnant PLWH-ART in DRC and globally. Pregnant PLWH-ART were predominantly classified as CST III and IV, which differ in predicted function. We also identified associations between systemic immunity and the VMB, suggesting directions for future research on maternal and infant health. Our meta-analysis showed that the HIV-associated VMB and its predicted functions differ from those of PWoH, contrary to earlier reports. Larger follow-up studies in Kinshasa using shotgun metagenomics will enable genome-resolved analyses, improved functional inference, and assessment of strain-level differences linked to adverse birth outcomes. Moving beyond a taxonomy-only CST framework toward combined functional and taxonomic classification will improve understanding of VMB roles in health and disease.

## MATERIALS AND METHODS

### Study cohort and sample collection

This pilot study included 82 participants (40% of total consenting to vaginal swabs) enrolled in Kinshasa, DRC, nested within the larger in the Continuous Quality Intervention: Preventing Mother to Child Transmission (CQI-PMTCT) study, a longitudinal evaluation of the long-term ART outcomes in pregnancy (NCT03048669) (33). From October 2020 to May 2021, clinicians collected vaginal swabs and peripheral blood from participants in their second or third trimesters who could access care during the SARS-CoV-2 pandemic. Swabs were stored in DNA/RNA shield (ZymoResearch, R1108) and stored at −80°C until shipped to Columbus, OH. Plasma from peripheral blood was processed immediately and stored similarly (Fig. 1A).

We collected demographic data, including participant age, gestational age, ART regimen, gravidity, and socioeconomic status (SES) at enrollment. SES was categorized into quartiles using PCA, with 0 as the lowest and three as the highest (117). Adverse birth outcomes were recorded at delivery.

### Study selection, search strategy, and criteria for meta-analysis

16S rRNA gene VMB studies were selected by conducting a thorough and manually curated search of the Web of Science Core Collection of Thomson Reuters (WOS) and the European Nucleotide Archive (ENA) for studies published until December 2022. The search terms, hits, and justification for exclusion or inclusion are shown in Fig. S1. The inclusion criteria were as follows: (i) pregnant, healthy, or living with HIV; (ii) availability of raw data, and (iii) Illumina paired-end sequencing of the V3–V4 hypervariable region. Additional details are provided in Supplemental methods.

### DNA EXTRACTION, 16S rRNA GENE SEQUENCING, AND PROCESSING

Bacterial DNA from vaginal swabs in the CQI-PMTCT cohort was extracted using the Qiagen AllPrep DNA/RNA Mini kit (Qiagen, 80204). To reduce contamination, samples were extracted in a clean room, and all materials were treated with 70% ethanol, RNAse, and UV light for ~15 min before DNA extraction. No bands were detected on gels when examining the PCR products from reagent-only controls extracted in parallel with each batch.

Samples were sent to SeqCenter (https://www.seqcenter.com/) for amplification of the V3–V4 region using the 341F (5′-CCTACGGGDGGCWGCAG-3′) and 806R (5′-GACTACNVGGGTMTCTAATCC-3′) primers. 16S rRNA gene reads were then processed following the DADA2 SOP (118). Additional details on sample and data processing are described in Supplemental methods.

For the five selected studies for the meta-analysis, sequences were processed on a per-study basis as described for the CQI-PMTCT sequences. Additional details on sequence processing are described in Supplemental methods.

PICRUSt2 version v2.4.2 (84) was used to predict functional composition from the 16S rRNA gene sequences. Data were normalized based on the abundance of 16S rRNA gene copy numbers. Metagenome predictions were used to estimate KEGG pathways, and any unassigned KEGG IDs were mapped using KEGGREST v3.19 (119).

### Quantification of plasma cytokine, chemokine, and soluble factors (immune factors)

Levels of immune factors measured from maternal plasma were quantified using the LEGENDplex COVID-19 Cytokine Storm Panel 1 & 2 (BioLegend, 741095), LEGENDplex Human Proinflammatory Chemokine Panel 1 (BioLegend, 740984), and LEGENDplex Vascular Inflammation Panel 1 (BioLegend, 740551). Additional details related to the measurement of immune factors are described in Supplemental methods.

### Statistical analyses

CSTs were assigned to each sample using VALENCIA (120). Alpha diversity indices (Chao1, Simpson, and Shannon) were estimated using an asymptotic statistical approach (121, 122). Differences in alpha diversity were tested using the Kruskal-Wallis test, followed by Wilcoxon pairwise comparison tests if significant.

Beta diversity analyses were computed using PCA on Robust Aitchison distances. Significance of PCAs was measured using ANOSIM from the vegan package v2.6-10. To identify the associations between VMB metrics (alpha diversity and bacterial abundance) and the log-transformed plasma immune factors concentration, linear regression models were computed using two-sided Spearman’s rank correlation. Adjustments for multiple hypothesis testing were not performed due to this being an exploratory study (123).

For the meta-analysis, batch effects were accounted for using PLSDA batch after evaluation of several batch removal methods (see Supplemental methods for more details). Alpha diversity indices and beta diversity on Aitchison distances were computed as above.

To identify differentially abundant species and predicted functions, three statistical methods were used: LinDA (v.1.1 (124, 125), ANCOM-BC v.2.2.1 (126), and ALDEx2 v.1.32.0 (127). A species was considered differentially abundant if it met two of the following criteria: *P*-adjusted value < 0.05 in LinDA; q-value < 0.05 in ANCOM-BC; or effect size > 0.5 in ALDEx2. Additional details on differential abundance analysis are described in Supplemental Methods. The strengthening the organization and reporting of microbiome studies (STORMS) checklist (128) (Table S5) is included for standardization and reproducibility of data.

## Data Availability

Raw sequence data files are available in the Sequence Read Archive (SRA) under BioProject PRJNA1234224; scripts and data used to analyze all the data are available on https://github.com/SulliVagOSU/16S_meta_analysis.

## SUPPLEMENTAL MATERIAL

Supplemental material msphere.00597-25-s0001.pdf Supplemental methods and figures.

Supplemental Tables msphere.00597-25-s0002.xlsx Tables S1-S9.

ASM does not own the copyrights to Supplemental Material that may be linked to, or accessed through, an article. The authors have granted ASM a non-exclusive, world-wide license to publish the Supplemental Material files. Please contact the corresponding author directly for reuse.
