# Supplementary material for: The vaginal microbiome of pregnant people living with HIV on antiretroviral therapy in the Democratic Republic of Congo: a pilot study and global meta-analysis
Source: mSphere. 2026 Jan 26;11(2):e00597-25. doi: 10.1128/msphere.00597-25 (PMC12931276; doi:10.1128/msphere.00597-25)
Supplement: Supplemental material — Supplemental methods and figures. [file msphere.00597-25-s0001.pdf]

Supplementary Materials for:

The cervicovaginal microbiome of pregnant people living with HIV on antiretroviral therapy in the Democratic Republic of Congo: A Pilot Study and Global Meta-analysis.

Kimberley S. Ndlovu[1,2,4*], Ricardo R. Pavan[1,2,4*], Jacqueline Corry[1,3,4], Ann C. Gregory[5] Samia Mahamed[1], Natalia Zotova[6], Martine Tabala[7], Pelagie Babakazo[7], Nicholas T. Funderburg[8], Marcel Yotebieng[6], Nichole R. Klatt[9], Jesse J. Kwiek[1,3,4#], & Matthew B. Sullivan[1,2,4,10#]

Correpsonding authors:

Matthew B. Sullivan sullivan.948@osu.edu

Jesse J. Kwiek kwiek.2@osu.edu

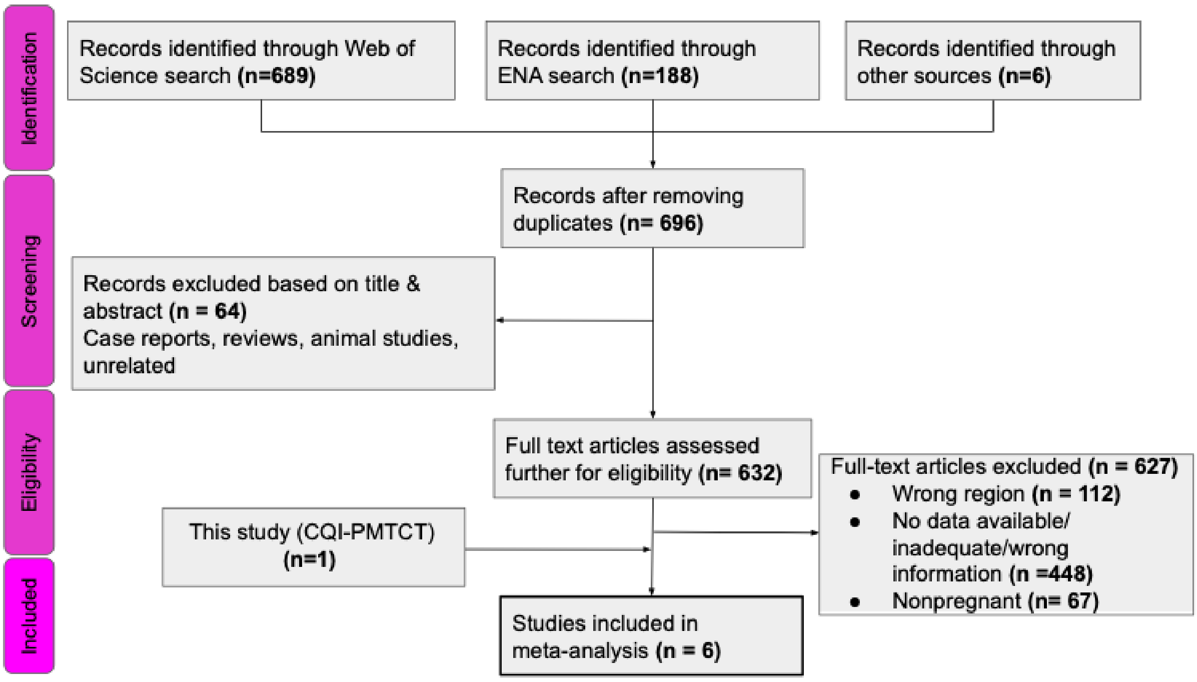

Supplemental Figure 1: PRISMA flowchart on selection of studies for meta-analysis. The search terms for ENA were: “vaginal microbiome” and for WOS: “(ALL= (country name OR nationality) AND TS= (vaginal OR vagina OR vagin*) AND TS= (microbiota* OR microflora* OR bacteria* OR microbiome* OR flora* OR bacterial* OR bacteria* OR microorganism OR dysbiosis) AND TS=(16S) AND TS= (woman OR women))”

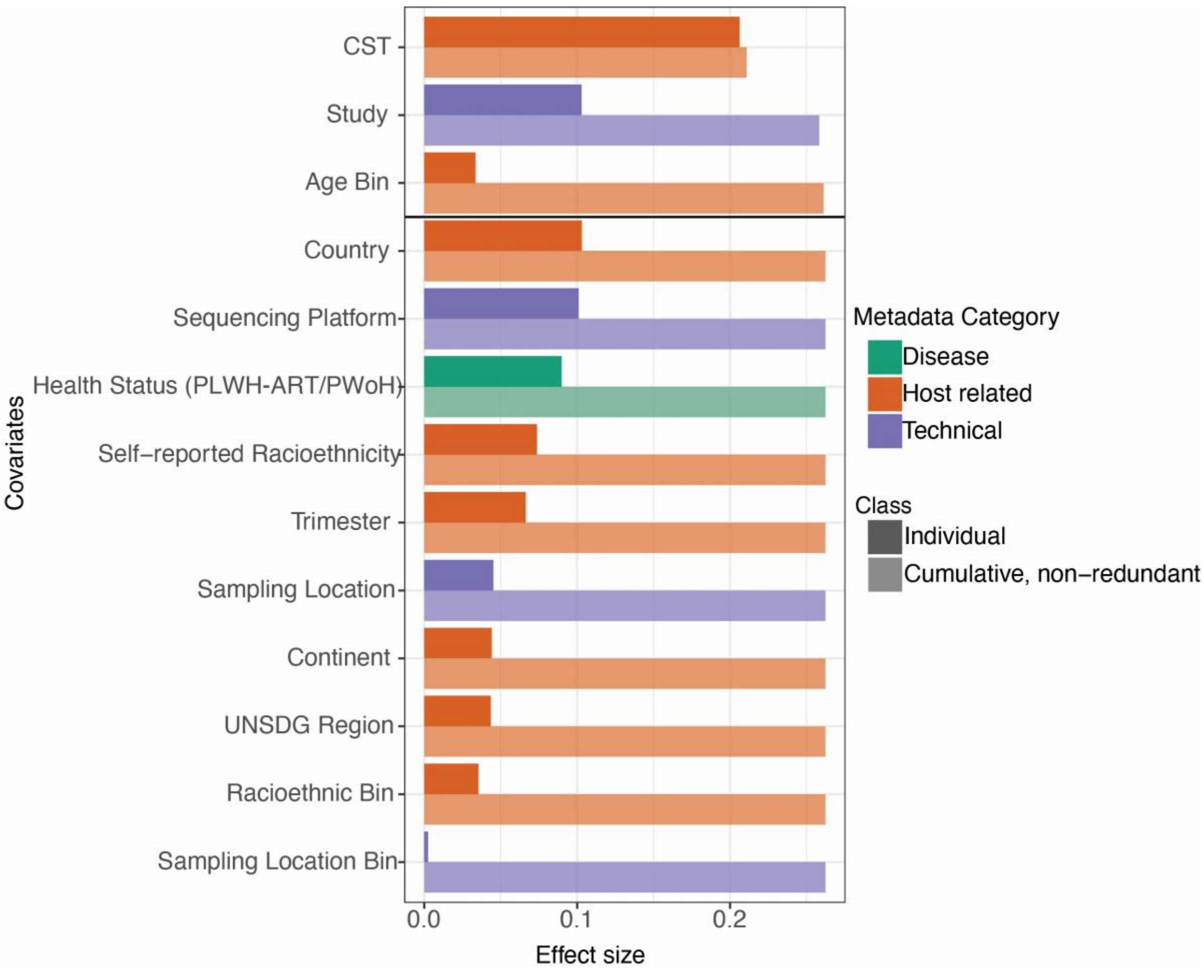

Supplemental Figure 2: Covariates explaining microbial variation in the CVMB of the 6 studies included in the meta-analysis (n=625, dbRDA). Only significant covariates shown (BH-corrected $p < 0.05$). Dark colors indicate the individual variance explained by each covariate and lighter colors show the cumulative, non-redundant variance explained by the covariates (n =625, stepwise dbRDA). Covariates above the black line represents those that were significant in the cumulative, non-redundant analysis .

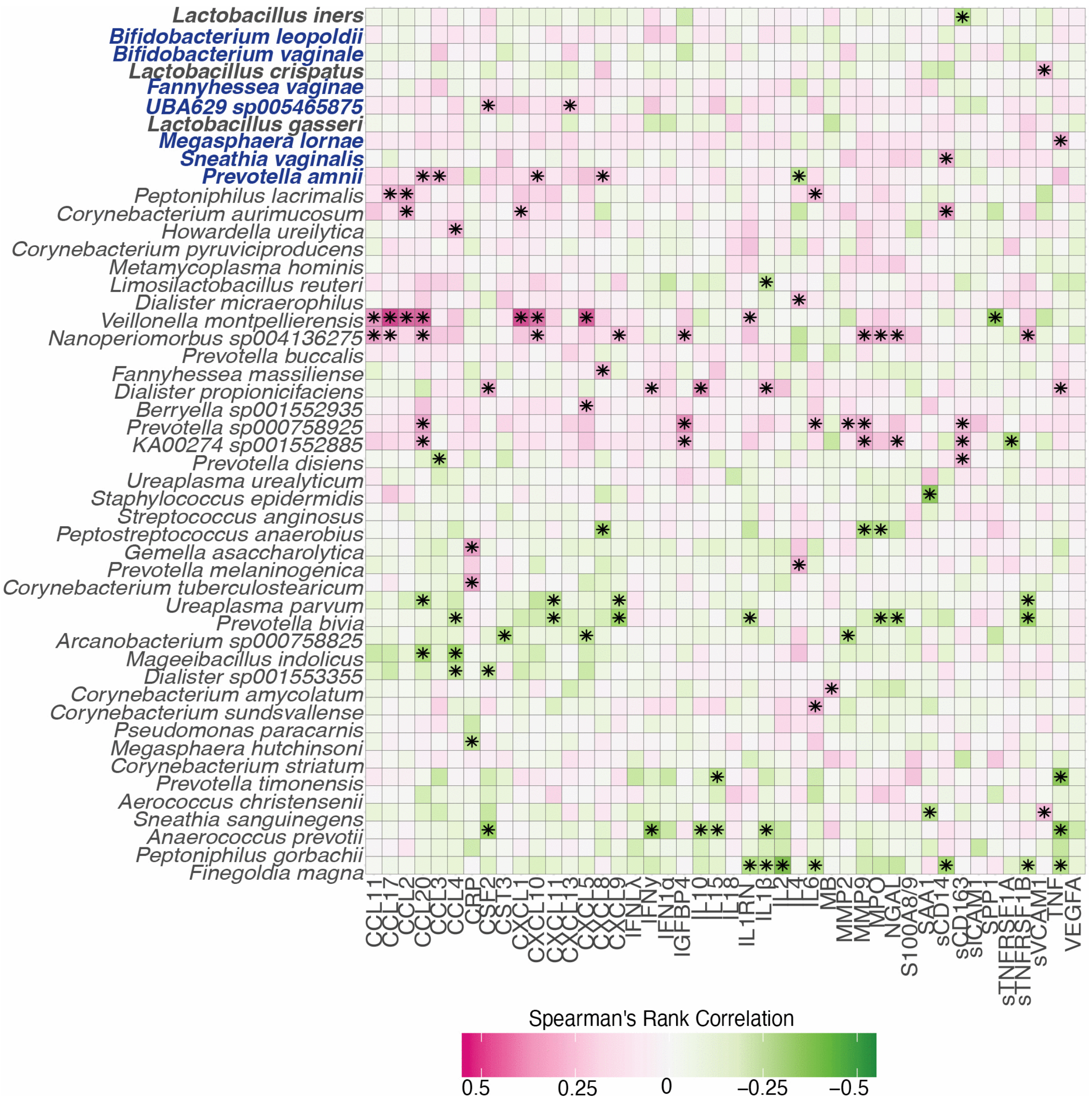

Supplemental Figure 3: Heatmap showing Spearman correlations between taxa and $\log_{10}$-normalized concentrations of immune factors. The top 10 most abundant taxa are bolded, and CST IV associated bacteria are highlighted in blue. Taxa are ordered by their overall abundance. Asterisk indicates unadjusted two sided spearman's $p < 0.05$.

Supplemental Methods

16S data sequencing and processing

Samples were sent to SeqCenter (https://www.seqcenter.com/) for amplification of the V3-V4 hypervariable region of the 16S rRNA gene using the 341F (5'-CCTACGGGDGGCWGCAG-3') and 806R (5'-GACTACNVGGGTMTCTAATCC-3') primers using the Zymo Research's Quick-16S kit. The V3-V4 region was chosen as it has been shown to identify the most taxa in vaginal samples (1). The PCR products were sequenced on an Illumina NextSeq2000 to generate 2x301bp paired-end reads.

16S rRNA gene reads were processed following the DADA2 SOP (2). Sequence quality was assessed using FASTQC v0.11.5 and any adapters or primer sequences were trimmed using Trimmomatic v0.36 (3, 4). Generally, sequences were trimmed at the first base with a quality score of Q<25. Taxonomy was assigned using the Greengenes2 database (5). Sequences were filtered to remove sequences of mitochondria and chloroplast, samples with less that 100 reads and taxa with less than 10 reads, less than 0.5% abundance across all samples and not present more than 2 times in 10% of samples were removed. The resultant ASV table was agglomerated to species-level and robust centered log-ratio (RCLR) transformation was performed on the table using the decostand function on the vegan package v2.6-6. This transformation was to mitigate the inherent negative correlation bias in compositional data (6, 7) The transformed table was used in all subsequent analyses.

For the studies selected for the meta-analysis, generated ASV tables per study were combined and taxonomy was assigned on the combined table (34,871 ASVs and 1861 samples). The aggregated table was further filtered by removing ASVs not assigned to any taxonomic level. To reduce data complexity, bacterial ASVs were filtered if observed at

frequencies of 0.0005% study-wide and observed in only 2 samples (8). Samples with fewer than 2000 reads or with excess zeroes were removed using the PreFL function from the PLSDAbatch package v1.0 (9). After filtering, ASVs were agglomerated at the species level resulting in an abundance table with 74 species-level taxa and 664 samples. A centered log-ratio (CLR) transformation was performed on the abundance table. Before the CLR transformation, zeros were replaced with a constant value smaller than the detection limit (e.g., 65% of the detection limit) (10, 11).

Study selection, search strategy and criteria for meta-analysis

Only studies with sufficient information for comparison were included (i.e., metadata and minimally reproducible 16S rRNA gene sequence processing pipeline). Metadata from each study was obtained from NCBI, and/or via correspondence with the authors of the selected studies. Among these studies, one was from Italy (12) and two from Finland (13, 14), one from the United States (15), and one from Uganda (16). Only the Ugandan study included samples from PLWH-ART (n = 5). More details on the studies can be found in **Table S1.**

Quantification of plasma cytokine, chemokine and soluble factors (immune factors).

All immune factor names, abbreviations and measurement units (ng/ml or pg/ml) can be found in **Supplemental Table 3**. Names and abbreviations were curated from uniport.org, *homo sapiens*. Names and abbreviations were curated from uniport.org, *homo sapiens,* following the International Protein Nomenclature Guidelines (17). Thirteen chemokines were simultaneously measured in plasma using LEGENDplex

Human Proinflammatory Chemokine Panel 1 (BioLegend, 740984) (interleukin-8 (IL-8), C-X-C motif chemokine 10 (CXCL10), eotaxin (CCL11), C-C motif chemokine 17 (CCL17), CCL2, CCL5, CCL3, CXCL9, CXCL5, CCL20, CXCL1, CXCL11, CCL4) according to the manufacturer's filter plate protocol. Similarly, twelve cytokines were measured in plasma using the LEGENDplex COVID-19 Cytokine Storm Panel 1 & 2 (BioLegend, 741095) (IL-6, interferon alpha-2 (IFN-α-2), IL-2, IFN-ɣ, IL-1RN, tumor necrosis factor (TNF- α), IL-10, granulocyte-macrophage colony-stimulating factor (GM-CSF), IL-1β, vascular endothelial growth factor a, long form (L-VEGF), IL-18, IL-15). Thirteen cytokines and soluble factors were measured in plasma using the LEGENDplex Vascular Inflammation panel 1 (BioLegend, 740551) (myoglobin (MB), protein S100-A8/protein S100-A9 (S100A8/S100A9), neutrophil gelatinase-associated lipocalin (NGAL) , c-reactive protein (CRP), 72 kDa type IV collagenase (MMP-2), osteopontin (SPP1), myeloperoxidase (MPO), serum amyloid A-1 protein (SAA), insulin-like growth factor-binding protein 4 (IGFBP4), soluble intercellular adhesion molecule 1 (sICAM1), soluble vascular cell adhesion protein 1 (sVCAM1), matrix metalloproteinase-9 (MMP-9), cystatin-C (CST3). Data were collected on the MACS Quant 10 Flow cytometer (Miltenyi Biotech) and using MacsQuant Software (Miltenyi Biotech). Gates were set around beads to exclude debris, and number of events collected was 300 per analyte multiplied by 1.1. Gates around individual bead sets and the corresponding gates around individual analytes were adjusted post collection in the LEGENDplex online Qognit software based on standards; gates were then applied to all standards and samples for a particular plate and five-parameter logistic standard curves were generated for all. Concentrations were interpolated from these curves using the LEGENDplex Qognit software (BioLegend, Qognit Inc.).

Seven immune factors were individually measured by duoset ELISA (R&D Systems): CXCL13 (DY801), IL-4 (DY204), IFN-λ-1(DY7246), soluble monocyte differentiation antigen CD14 (sCD14, DY383), soluble scavenger receptor cysteine-rich type 1 protein M130 (sCD163, DY1607), soluble tumor necrosis factor receptor superfamily member 1A (sTNFRSF1A, DY225), sTNFRSF1B (DY726) according to the manufacturer's instructions with minor modifications. First half-well plates were used (Greiner, 675061), plates were washed using the CAPP wash 12 (Pipette.com, W-12) attached to a carboy that was washed daily, and plates were developed using TMB substrate Plus liquid (VWR, 97063-666). Absorbances were read on a SpectraMax i3x (Molecular Devices).

For all immune factor assays performed there were three samples that were on all plates to serve as quality control to ensure similar assay performance across plates and days. See **Supplemental Table 4** for dilution factors and upper and lower limits of detection (LoD). Values below the limit of detection or below the bottom standard were increased to the bottom standard, unless the dilution factor was >2, then the bottom standard was multiplied by the dilution factor. Values above the limit of detection were replaced by the top standard multiplied by the dilution factor multiplied by 1.1 (**Supplemental Table 4**).

Statistics

To avoid batch effects, removeBatchEffect (rBE from the limma package) (18), ComBat from the sva package in R (19), Partial Least Square Discriminant Analysis (PLSDA-batch), sparse PLSDA (sPLSDA-batch), weighted PLSDA (wPLSDA-batch), sparse weighted PLSDA (wPLSDA-batch), from the PLSDAbatch package, and batch-

mean centering transformation (BMC). To evaluate batch adjustment correction the following methods were used: principal component analysis (PCA); heatmap and cluster analysis using tidyHeatmap v. 1.8.1 (20); alignment score (PLSDAbatch package); and partial redundancy analysis (pRDA) using vegan v. 2.6-4 (21).

To identify differentially abundant species and predicted functions, three statistical methods were used: LinDA from the MicrobiomeStat package v.1.1 (22, 23) , ANCOM-BC v.2.2.1(24) and ALDEx2 v.1.32.0 (25) with the formula "abundance ~ health_status + study". A species was considered differentially abundant if it met two of the following criteria: p-adjusted value < 0.05 in LinDA; q-value < 0.05 in ANCOM-BC; or effect size > 0.5 in ALDEx2. A score was computed to indicate the number of methods that identified a species as differentially abundant. For example, if a species was identified by both LinDA (p-adjusted value < 0.05) and ALDEx2 (effect size > 0.5), it received a score of 2. This combination of methods aims to overcome drawbacks such as low power and high false discovery rate (FDR) in differential abundance methods (26). The input was the count table without CLR transformation. In LinDA, a heuristic imputation method was used, with imputed values proportional to library sizes. ANCOM-BC and ALDEx2 included their own transformations and bias corrections. The analysis was conducted using default arguments for all methods.

References

1. Graspeuntner S, Loeper N, Künzel S, Baines JF, Rupp J. 2018. Selection of validated hypervariable regions is crucial in 16S-based microbiota studies of the female genital tract. Sci Rep 2018 81 8:1–7.

2. Callahan BJ, McMurdie PJ, Rosen MJ, Han AW, Johnson AJA, Holmes SP. 2016. DADA2: High-resolution sample inference from Illumina amplicon data. Nat Methods 13.
3. Andrews S. 2010. FastQC: A quality control analysis tool for high throughput sequencing data. https://www.bioinformatics.babraham.ac.uk/projects/fastqc/. Retrieved 12 October 2022.
4. Bolger AM, Lohse M, Usadel B. 2014. Trimmomatic: a flexible trimmer for Illumina sequence data. Bioinformatics 30:2114–2120.
5. McDonald D, Jiang Y, Balaban M, Cantrell K, Zhu Q, Gonzalez A, Morton JT, Nicolaou G, Parks DH, Karst SM, Albertsen M, Hugenholtz P, DeSantis T, Song SJ, Bartko A, Havulinna AS, Jousilahti P, Cheng S, Inouye M, Niiranen T, Jain M, Salomaa V, Lahti L, Mirarab S, Knight R. 2023. Greengenes2 unifies microbial data in a single reference tree. Nat Biotechnol 2023 14:1–4.
6. Aitchison J, Egozcue JJ. 2005. Compositional Data Analysis: Where Are We and Where Should We Be Heading? Math Geol 37.
7. Gloor GB, Macklaim JM, Pawlowsky-Glahn V, Egozcue JJ. 2017. Microbiome datasets are compositional: And this is not optional. Front Microbiol 8:294209.
8. Cao Q, Sun X, Rajesh K, Chalasani N, Gelow K, Katz B, Shah VH, Sanyal AJ, Smirnova E. 2021. Effects of Rare Microbiome Taxa Filtering on Statistical Analysis. Front Microbiol 11:607325.

9. Wang Y, Lê Cao K-A. 2023. PLSDA-batch: a multivariate framework to correct for batch effects in microbiome data. Brief Bioinform 24:bbac622.

10. Bastiaanssen TFS, Quinn TP, Loughman A. 2023. Bugs as Features (Part II): A Perspective on Enriching Microbiome-Gut-Brain Axis Analyses. Nat Ment Health 1:930–938.

11. Lubbe S, Filzmoser P, Templ M. 2021. Comparison of zero replacement strategies for compositional data with large numbers of zeros. Chemom Intell Lab Syst 210:104248.

12. Severgnini M, Morselli S, Camboni T, Ceccarani C, Laghi L, Zagonari S, Patuelli G, Pedna MF, Sambri V, Foschi C, Consolandi C, Marangoni A. 2022. A Deep Look at the Vaginal Environment During Pregnancy and Puerperium. Front Cell Infect Microbiol 12.

13. Kervinen K, Holster T, Saqib S, Virtanen S, Stefanovic V, Rahkonen L, Nieminen P, Salonen A, Kalliala I. 2022. Parity and gestational age are associated with vaginal microbiota composition in term and late term pregnancies. eBioMedicine 81:104107.

14. Livson S, Virtanen S, Lokki AI, Holster T, Rahkonen L, Kalliala I, Nieminen P, Salonen A, Meri S. 2022. Cervicovaginal Complement Activation and Microbiota During Pregnancy and in Parturition. Front Immunol 13:925630.

15. Ho M, Moon D, Pires-Alves M, Thornton PD, McFarlin BL, Wilson BA. 2021. Recovery of microbial community profile information hidden in chimeric sequence reads. Comput Struct Biotechnol J 19:5126–5139.

16. Movassagh M, Bebell LM, Burgoine K, Hehnly C, Zhang L, Moran K, Sheldon K, Sinnar SA, Mbabazi-Kabachelor E, Kumbakumba E, Bazira J, Ochora M, Mulondo R, Nsubuga BK, Weeks AD, Gladstone M, Olupot-Olupot P, Ngonzi J, Roberts DJ, Meier FA, Irizarry RA, Broach JR, Schiff SJ, Paulson JN. 2021. Vaginal microbiome topic modeling of laboring Ugandan women with and without fever. Npj Biofilms Microbiomes 7:1–10.

17. National Center for Biotechnology Information (NCBI), European Bioinformatics Institute, (EMBL-EBI), Protein Information Resource (PIR), Swiss Institute for Bioinformatics (SIB). 2020. International Protein Nomenclature Guidelines. https://www.ncbi.nlm.nih.gov/genbank/internatprot_nomenguide/. Retrieved 28 March 2024.

18. Ritchie ME, Phipson B, Wu D, Hu Y, Law CW, Shi W, Smyth GK. 2015. limma powers differential expression analyses for RNA-sequencing and microarray studies. Nucleic Acids Res 43:e47.

19. Leek JT, Johnson WE, Parker HS, Jaffe AE, Storey JD. 2012. The sva package for removing batch effects and other unwanted variation in high-throughput experiments. Bioinformatics 28:882–883.

20. Mangiola S, Papenfuss AT. 2020. tidyHeatmap: an R package for modular heatmap production based on tidy principles. J Open Source Softw 5:2472.

21. Oksanen J, Blanchet FG, Kindt R, Legendre P, Minchin P. 2007. The vegan package: Community Ecology Package. R package version 2.0–2.

22. Lu Y, Zhou G, Ewald J, Pang Z, Shiri T, Xia J. 2023. MicrobiomeAnalyst 2.0: comprehensive statistical, functional and integrative analysis of microbiome data. Nucleic Acids Res 51:W310–W318.

23. Zhou H, He K, Chen J, Zhang X. 2022. LinDA: linear models for differential abundance analysis of microbiome compositional data. Genome Biol 23:95.

24. Lin H, Peddada SD. 2020. Analysis of compositions of microbiomes with bias correction. Nat Commun 11:3514.

25. Fernandes AD, Macklaim JM, Linn TG, Reid G, Gloor GB. 2013. ANOVA-Like Differential Expression (ALDEx) Analysis for Mixed Population RNA-Seq. PLOS ONE 8:e67019.

26. Hawinkel S, Mattiello F, Bijnens L, Thas O. 2019. A broken promise: microbiome differential abundance methods do not control the false discovery rate. Brief Bioinform 20:210–221.
